# Complicated Rheumatoid Nodules in Lung

**DOI:** 10.1155/2020/6627244

**Published:** 2020-12-02

**Authors:** Geetha Wickrematilake

**Affiliations:** Sirimavo Bandaranayake Specialized Childrens Hospital, Kandy, Sri Lanka

## Abstract

A 65-year-old nonsmoker lady carrying a diagnosis of seropositive erosive rheumatoid arthritis for nine years presented with acute shortness of breath, following a spontaneous pneumothorax while on combination therapy with methotrexate, leflunomide, and tocilizumab. Imaging studies revealed multiple cavitory lung nodules, and a transbronchial lung biopsy favoured a diagnosis of rheumatoid lung nodules. Her initial pathological samples were negative for any infectious cause. A follow-up computerized tomography scan (CT scan) confirmed enlargement of lung nodules with a positive antibody test for aspergillosis which needed antifungal therapy, and currently, her arthritis is managed well with rituximab therapy, sulfasalazine, and hydroxychloroquine.

## 1. Introduction

Pulmonary rheumatoid nodules are a rare complication of rheumatoid arthritis, and complications of rheumatoid nodules are scant in literature.

Here is a case of RA without cutaneous rheumatoid nodules who presented with complicated rheumatoid lung nodules while on immunosuppressive therapy.

## 2. Case Presentation

A 65-year-old nonsmoker lady, with a history of seropositive erosive rheumatoid arthritis for nine years, presented with acute onset of shortness of breath. There was no previous history of cough, shortness of breath, fever, malaise, or weight loss. She denied any history of recent travel and denied any contact with sick patients. There was no history of occupational or environmental exposures.

The patient was on monthly tocilizumab infusions, methotrexate 20 mg weekly, and leflunomide 20 mg/day for her rheumatoid arthritis.

On examination, there were no skin nodules or lymphadenopathy. However, she had joint tenderness with a Disease Activity Score-28 (DAS 28) of 5.9 on current presentation.

The oxygen saturation was 98% while breathing room air, but the chest expansion was reduced on the right side. Her chest x-rays revealed a pneumothorax on the right side and multiple lung nodules.

**Figure 1:**
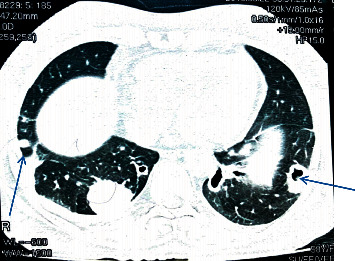
CT scan of lungs showing rheumatoid lung nodules.

**Figure 2:**
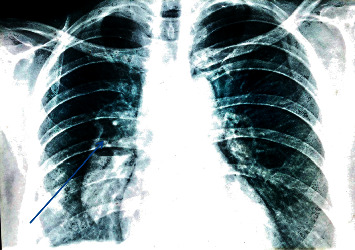
CXR showing lung nodules.

After treating the pneumothorax with an intercostal tube, she was investigated for lung nodules and a CT scan confirmed multiple thick-walled cavitory lung nodules in both lung fields with no evidence of bronchiectasis (Figures 1 and 2). Adjacent lung and the mediastinum were normal. However, an irregular soft tissue density was filling some of the nodules.

She had normal full blood count and basic metabolic panel levels. The patient's rheumatoid factor was 256 U/ml, and anti-CCP was >200 U/ml. c and p antineutrophil cytoplasmic antibodies (ANCA) were negative with an ESR of 110 mm/hr and CRP of 130 mg/dL. Echocardiogram was normal with negative blood cultures. Her ultrasound abdomen examination was negative with no evidence of malignancy.

Sputum tests for acid-fast bacillus (AFB) were negative thrice, and bronchoalveolar lavage fluid revealed few scattered polymorphs with no malignant cells. It was negative for AFB, and fungal stains and cultures for AFB were negative. Her Mantoux was 5 mm. Transbronchial lung biopsy evidenced a collection of macrophages, lymphocytes, and plasma cells around an area of necrosis, with no evidence of malignancy.

Her arthritis was maintained with prednisolone, hydroxychloroquine (HCQ), and sulfasalazine (SSZ).

A repeat CT scan done 6 months later showed expansion of her lung cavities, and an aspergillus precipitin test (immunoglobulin G (IgG)) became positive and treatment was started for chronic pulmonary aspergillosis.

She was treated with itraconazole 300 mg bd, for 9 months for the fungal infection, and was treated with sulfasalazine 1 g bd and hydroxychloroquine 200 mg daily for the arthritis while steroids were tailed off to a maintenance dose of 7.5 mg/day.

Once her lung condition was stabilized, she was treated with rituximab 1g two weeks apart with good control of her arthritis (DAS-28 was 1.9) at four months. She is still under shared care between the rheumatologist and the chest physician. However, follow-up CT scan done after 1 year has not shown any significant change in nodule size.

Pneumothorax and fungal infection in pulmonary rheumatoid nodules were very rare in the literature and the patient's arthritis is controlled with rituximab therapy.

## 3. Discussion

The differential diagnosis of cavitary pulmonary nodules in patients with rheumatoid arthritis includes infections (bacterial infections including septic emboli, fungal, and mycobacterial), malignancies (primary or metastatic), vasculitides (Wegener's) drug reactions, and rheumatoid nodules.

Embolic disease is unlikely in this patient given her normal echocardiogram and blood cultures, but other types of infection have to be seriously considered given her state of immunosuppression. Her ANCA levels were negative, and there were no features of vasculitis.

Reactivation of tuberculosis (*M*. *tuberculosis*) (TB) involves the upper lobes, while primary TB usually occurs as a lower lobe disease. There is an increased incidence of TB reactivation in recipients of biologics. This patient's sputum and samples of bronchoalveolar lavage were negative for AFB and fungi. Her tuberculosis screen with Mantoux was 5 mm, which was considered to be a false-positive result from BCG vaccination (≥5 mm considered positive in patients on immunosuppressant therapy), TB cultures were negative, and culture for fungal studies and the transbronchial lung biopsy revealed granuloma formation with areas of necrosis with surrounding inflammatory infiltrates and histiocytic proliferation, consistent with necrotizing granulomatous inflammation.

Pathological examination of rheumatoid nodules shows central fibrinoid necrosis with palisading mononuclear cells and associated vasculitis, and our patient's biopsy pathology had similar features.

Pulmonary rheumatoid nodules are an uncommon extra-articular manifestation of RA (prevalence < 0.4%–32% depending on the mode of investigation) [1]. They occur in patients with longstanding disease and subcutaneous nodules and are typically located along the interlobular septa or in subpleural regions [2]. They predominate in men (7 : 1 ratio) and appear late in the course of RA [3]. They are rarely symptomatic, although they can present with cough and hemoptysis. Usually, these regress with standard DMARD therapy but paradoxically may enlarge in size.

Pulmonary nodulosis has been shown to be accelerated by methotrexate, and nodules are induced by leflunomide, azathioprine, and antitumor necrosis factor (anti-TNF) etanercept, and infliximab [4–7].

Marked improvement of rheumatoid lung nodules has been shown after treatment with tocilizumab, while in one case series, tocilizumab has been shown to increase subcutaneous nodules [8, 9].

Rituximab has been shown to regress pulmonary nodules in a retrospective study [10].

Up to 50% of rheumatoid nodules may cavitate or lead to an associated pleural effusion, pneumothorax ,or hydropneumothorax. Fungus colonisation is a rare complication of rheumatoid nodules [11].

Spontaneous pneumothorax is a rare but well-recognised complication of rheumatoid nodule, probably secondary to a bronchopleural fistula [12].

Usually, pulmonary lung nodules occur in patients with seropositive RA who were on long-term therapy. Hence, both MTX and leflunomide may have contributed to the formation of lung nodules in this patient. However, she was also on tocilizumab which is beneficial for patients with lung nodules [8].

After discontinuation of disease-modifying anti-rheumatic drugs (DMARDS), the patient had only few options to control her rheumatoid arthritis. Rituximab, a drug shown to be beneficial for patients with rheumatoid nodules, was tried with two other conventional synthetic DMARDS (SSZ and HCQ), and now, the patient is out of systemic steroids with satisfactory control of her arthritis. Her lung problem is periodically monitored by a chest physician. But lung nodules have not shown any regression and persist as the same.

So in conclusion, rheumatoid lung nodules may fluctuate with the course of the disease or may be related to drugs used to manage RA. Making an accurate diagnosis is very important as malignancies and infections may also have similar appearance on imaging. Lung biopsy is mandatory to confirm the histopathological features of the nodule. Treatment will be changing the basal treatment of RA to another one that has been documented to cause lesser damage to the lung tissue. Surgery may also be required when there is high risk of nodule rupture, hemorrhage, or tension pneumothorax with a potential for increased patient mortality.

## Data Availability

Patient investigation reports used to support the findings of this study are available from the corresponding author upon request.

## Additional Points

*Summary.* Rheumatoid lung nodules are a rare pulmonary manifestation of rheumatoid arthritis (RA).Usually, they are asymptomatic and occur in seropositive long-standing RA patients. A biopsy is needed for diagnosis.They can worsen with therapy, especially methotrexate, leflunomide, azathioprine, and TNF inhibitors. Patients with rheumatological diseases, particularly those receiving high-dose immunosuppressive drugs, are at increased risk of life-threatening aspergillus infections.
